# Notes and News

**Published:** 1901-04-27

**Authors:** 


### NOTES AND NEWS.

Hong Kong is on the black list again, having to record cases of plague, cholera, and smallpox.

The new Richmond Surgical Hospital, Dublin, was opened last week. Very great care has been taken in the planning and details of the building. The operating theatre has been made a great feature. It is ventilated on the Plenum system. Messrs. Carroll & Batchelor, 86 Merrion Square, Dublin, are the architects.

We regret that the name of Dr. W. P. Mears was given last week as one of the medical officers of the Woodburn Sanatorium for Consumption, Morningside, Edinburgh. As a matter of fact, Dr. W. P. Mears died recently, and his widow, a duly qualified doctor, has taken over the charge of the establishment as senior physician.

The following honours have been bestowed amongst the medical profession for services in South Africa:—Sir William MacCormac has been created a K.C.M.G., Sir Thomas Naghten Fitzgerald, Sir William Thomson, Messrs. Alfred Fripp, G. Makins, F. Treves, Watson Cheyne, G. L. Cheatle, Kendal Franks, and J. Chiene have been made Commanders of the Order of the Bath.

Lady Sarah Wilson desires to express to the English public her most grateful thanks for the truly liberal manner in which they have responded to her appeal for books for the South African Constabulary. No less than twenty-two tons of literature have been received at Delahay Street since the beginning of April, being ample for immediate requirements. Newspapers may be sent to Headquarters, South African Constabulary, South Africa direct, where they will be most welcome. Any further communications respecting books already dispatched, or on the way, should be addressed to Mr. Oliver Williams, 71 and 72 King William Street, London, E.C.

The most stringent laws are being passed against opium smoking in Korea.

Yellow Fever has broken out on board H.M.S. *Condor*, which is in quarantine at Victoria, British Columbia.

Plague cases made their appearance in March in the Australian capitals of Perth, Adelaide, Sydney, and Brisbane.

The festival dinner of the Royal Hospital for Diseases of the Chest will be held at the Whitehall Rooms, Hôtel Métropole, on Monday, May 6th, Sir Thomas A. de la Rue presiding.

St. Luke's Hospital, the new workhouse hospital at Halifax, has been opened by Sir J. Crichton Browne. The present accommodation is for 400 beds, which have been provided at a cost of £98,000, being £5,000 lower than the estimates allowed by the Local Government Board.

The income of the Worcester Infirmary has become so much smaller during the last few years that it has been decided to close a ward. We are afraid that unless this drastic step stimulates the public to support the infirmary more liberally, it will not be a very effectual means of removing the annual deficit. When the establishment is in full working order, the extra cost of maintaining a ward is not very great.

Dr. Rolleston, a chef, and 32 ward orderlies left Waterloo on Saturday for the Cape in the hospital ship *Dunera*, thence to proceed to the Imperial Yeomanry Hospital at Pretoria. This detachment is taking out a quantity of medical comforts, warm clothing, books, and other presents which have been received for the patients in the hospital; also three cases (5 cwt.) of essence of cocoa, a gift from Messrs. Cadbury Brothers, Birmingham.

The battle as to whether the Royal Orthopædic Hospital shall rebuild on its present site, or remove to a less expensive locality, is still waging between some of the management and the medical staff. From a standpoint of common sense we should have thought there could not be two opinions. Seeing that the Orthopædic is not an emergency hospital, there is no reason that we can see for sacrificing the large sum of money which will accrue to it if the present expensive site is sold, merely to keep it in one of the busiest of thoroughfares, and possibly within easy distance of residential medical quarters. The question of the applying public funds to the best advantage is one that is apt to be put aside.

We learn from Australia that in spite of the efforts of legislators to stamp out private lunatic asylums, "the law is still evaded," and "medical homes" are still to be found in Australia, where the relatives of someone "peculiar," but who has not been certified as a person of unsound mind, can be placed. Some of these homes are quite luxurious in their appointments and very well managed, and what the official mind seems unable to grasp is, that all people do not like to place their afflicted relatives in one of the great overcrowded public caravanserais for the insane. However, in Victoria, when once an individual gets the label of unsound mind attached to his name, he must go into one of these institutions.

